# Delivery of the autofluorescent protein R-phycoerythrin by calcium phosphate nanoparticles into four different eukaryotic cell lines (HeLa, HEK293T, MG-63, MC3T3): Highly efficient, but leading to endolysosomal proteolysis in HeLa and MC3T3 cells

**DOI:** 10.1371/journal.pone.0178260

**Published:** 2017-06-06

**Authors:** Mathis Kopp, Olga Rotan, Chrisovalantis Papadopoulos, Nina Schulze, Hemmo Meyer, Matthias Epple

**Affiliations:** 1 Inorganic Chemistry and Center for Nanointegration Duisburg-Essen (CeNIDE), University of Duisburg-Essen, Essen, Germany; 2 Centre for Medical Biotechnology, Faculty of Biology, University of Duisburg-Essen, Essen, Germany; 3 Imaging Centre Campus Essen (ICCE), University of Duisburg-Essen, Essen, Germany; Chung-Ang University College of Engineering, REPUBLIC OF KOREA

## Abstract

Nanoparticles can be used as carriers to transport biomolecules like proteins and synthetic molecules across the cell membrane because many molecules are not able to cross the cell membrane on their own. The uptake of nanoparticles together with their cargo typically occurs via endocytosis, raising concerns about the possible degradation of the cargo in the endolysosomal system. As the tracking of a dye-labelled protein during cellular uptake and processing is not indicative of the presence of the protein itself but only for the fluorescent label, a label-free tracking was performed with the red-fluorescing model protein R-phycoerythrin (R-PE). Four different eukaryotic cell lines were investigated: HeLa, HEK293T, MG-63, and MC3T3. Alone, the protein was not taken up by any cell line; only with the help of calcium phosphate nanoparticles, an efficient uptake occurred. After the uptake into HeLa cells, the protein was found in early endosomes (shown by the marker EEA1) and lysosomes (shown by the marker Lamp1). There, it was still intact and functional (*i*.*e*. properly folded) as its red fluorescence was detected. However, a few hours after the uptake, proteolysis started as indicated by the decreasing red fluorescence intensity in the case of HeLa and MC3T3 cells. 12 h after the uptake, the protein was almost completely degraded in HeLa cells and MC3T3 cells. In HEK293T cells and MG-63 cells, no degradation of the protein was observed. In the presence of Bafilomycin A1, an inhibitor of acidification and protein degradation in lysosomes, the fluorescence of R-PE remained intact over the whole observation period in the four cell lines. These results indicate that despite an efficient nanoparticle-mediated uptake of proteins by cells, a rapid endolysosomal degradation may prevent the desired (*e*.*g*. therapeutic) effect of a protein inside a cell.

## Introduction

The transport of (bio-)molecules into cells is an ongoing issue in modern biomedical research. Many drug targets are located inside the cell [1], and the delivery of nucleic acids for gene delivery (DNA: transfection; siRNA and μRNA: gene silencing) requires the introduction of these biomolecules into a cell [2]. As many molecules, including proteins, are not able to cross the cell membrane on their own, suitable carriers are necessary [3]. Liposomes, dendrimers, polymers, and inorganic nanoparticles are prominent examples [4–12].

Prominent inorganic nanoparticles for drug delivery are noble metals [13, 14], iron oxide [15, 16], quantum dots [17, 18], and calcium phosphate [19–22]. Calcium phosphate nanoparticles have been used to transport different synthetic molecules and biomolecules like nucleic acids, proteins, or antigens across the cell membrane [20, 22–35]. They combine several advantages because they are already present in the body as mineral of human hard tissue (bone and teeth) [19]. Thus, they are highly biocompatible (unless the dose is very high, leading to a high intracellular calcium concentration) [36–39]. They are taken up by cells within a few hours [40], dissolved in the lysosome and finally excreted in ionic (*i*.*e*. dissolved) form [39]. As most other kinds of nanoparticles [8, 41–46], calcium phosphate nanoparticles are taken up by endocytosis, more specifically by micropinocytosis [47], leading to a delivery into endosomes and subsequently into lysosomes [47]. Generally, a degradation by an acidic environment and by proteases and nucleases occurs in lysosomes [48]. This has raised concerns about the fate of a biomolecule after cellular uptake because its integrity and function may be damaged after lysosomal processing [49–52].

Experiments where pathway and fate of a nanoparticle or a biomolecule are tracked are typically based on fluorescently labelled nanoparticles or biomolecules [53, 54]. For this, a fluorescent dye is attached either to the nanoparticle or to the biomolecule. However, this approach has its limitations as strictly speaking, only the dye is tracked and not the biomolecule. Neither a separation of the biomolecule from the nanoparticle nor the integrity and function of the biomolecule can be probed by this approach. Therefore, we have chosen a fluorescent protein (R-phycoerythrin) to realize a label-free uptake into cells, with and without the help of nanoparticles. This eliminates the need for fluorescent labelling, and also probes the functional integrity of the protein. Thus, the fate of the protein inside a cell can be followed. It also permits to track the pathway inside the cell after endocytotic uptake. Four commonly applied eukaryotic cell lines were used in this study: HeLa (human epithelial cell line), HEK293T (human epidermial cell line), MG-63 (human osteosarcoma cell line), and MC3T3 (mouse osteoblast cell line).

## Materials and methods

### Synthesis

The synthesis of calcium phosphate/polyethyleneimine/R-phycoerythrin (CaP/PEI/R-PE) nanoparticles was carried out as follows. Aqueous solutions of calcium nitrate (6.25 mM; Merck p.a.) and diammonium hydrogen phosphate (3.74 mM; Merck) were rapidly mixed by pumping them into a glass vessel with a peristaltic pump. The pH of both solutions was adjusted before with NaOH (0.1 M; Merck) to 9. A few seconds after mixing, 1 mL of the formed calcium phosphate nanoparticle dispersion was taken with a syringe and rapidly mixed with 0.2 mL of a polyethyleneimine solution (PEI; Sigma-Aldrich, MW 25 kDa; 2 mg mL^-1^) to achieve the colloidal stability of the nanoparticle dispersion. The positive zeta potential (+22 mV) indicates a successful stabilization with PEI.

1 mL of this dispersion was mixed with 1 mL of the dissolved protein phycoerythrin (R-PE; 1 mg mL^-1^) under thorough vortexing. The particles were separated from dissolved counter-ions and non-adsorbed molecules by centrifugation (21,000 g; 30 min; 4°C) with subsequent redispersion in the same volume of water with a sonotrode (Hielscher UP50H; sonotrode 3; cycle 0.8, amplitude 60%, 30 s). All inorganic salts were of p.a. quality. Ultrapure water (Purelab ultra instrument from ELGA) was used for all preparations. Phycoerythrin (R-PE from Rhodomonas) was obtained from Molecular Probes® by LifeTechnologiesTM (Eugene, Oregon, USA), dissolved at 4 mg mL^-1^ in ammonium sulphate/potassium phosphate buffer at pH 7.0, and used as obtained. All dilutions of this R-PE solution were done with pure water. For loading the calcium phosphate nanoparticles as described above, we have diluted R-PE with pure water to 1 mg mL^-1^. All syntheses were carried out at room temperature.

In the final dispersion, the calcium concentration as determined by atomic absorption spectroscopy was 5.0 μg mL^-1^. Assuming the stoichiometry of hydroxyapatite, Ca_5_(PO_4_)_3_OH, spherical particles (radius from SEM 75 nm), and the density of hydroxyapatite (3.14×10^3^ kg m^-3^), this corresponds to a particle concentration of 2.27×10^9^ particles mL^-1^, computed with the following parameters: Concentration of hydroxyapatite: 12.6 μg mL^-1^, volume of one particle: 1.77×10^−21^ m^3^, weight of one particle 5.54×10^−18^ kg. The amount of R-PE on the dispersed particles was determined by UV spectroscopy after removal of non-adsorbed protein by centrifugation. About 90% of the protein was adsorbed onto the particles, leading to a concentration of R-PE of 443 μg mL^-1^ in the nanoparticle dispersion. This corresponds to about 4.9×10^5^ R-PE molecules per nanoparticle, based on *M* = 2.4×10^5^ g mol^-1^, *m* = 3.99×10^−22^ kg per R-PE molecule, and 1.11×10^15^ R-PE molecules per mL. With a surface area of each nanoparticle of 7.07×10^−14^ m^2^ (70,700 nm^2^), each R-PE occupies about 0.14 nm^2^. This indicates that the loading of the nanoparticles with R-PE is rather high, exceeding a monolayer on the particle surface, probably by incorporation into the PEI polyelectrolyte shell. This stock solution of CaP/PEI/R-PE nanoparticles was used for all cell experiments.

### Characterization

Dynamic light scattering and zeta potential determinations were performed with a Zetasizer Nano series instrument (Malvern Nano-ZS, laser wavelength *λ* = 532 nm) using the Smoluchowski approximation and taking the data from the Malvern software without further correction. The particle size data refer to scattering intensity distributions (z-average). Scanning electron microscopy was performed with an ESEM Quanta 400 instrument (FEI), equipped with energy-dispersive X-ray spectroscopy (EDX; Genesis 4000, SUTW-Si(Li) detector) operating in a high vacuum with gold/palladium-sputtered samples. Centrifugation was performed at 4°C with a Heraeus Fresco 21 centrifuge. The amount of calcium was determined by atomic absorption spectroscopy (AAS) with an M-Series AA spectrometer (ThermoElectron, Schwerte). The concentration of nanoparticles in the dispersion was estimated using the calcium concentration as outlined below. The amount of R-PE on the nanoparticles was determined by quantitative UV spectroscopy, using a calibration curve at *λ* = 497 nm.

### Antibodies and reagents

Mouse anti-Lamp1 (sc-20011) was purchased from Santa Cruz Biotechnology. Mouse anti-EEA1 (610457) was obtained from BD Transduction Laboratories. Alexa Fluor® 633 secondary antibodies, Alexa Fluor® 660 phalloidin and DAPI were purchased from Thermo Fisher Scientific. Hoechst33342 and Bafilomycin A1 were obtained from Sigma.

### Cell culture

HeLa cells (human epithelial cervical cancer cells) were cultured in DMEM, supplemented with 10% fetal bovine serum (FBS) at 37°C (5% CO_2_, humidified atmosphere) according to standard cell culture protocols. HEK293T cells (human epidermal kidney cells) and MG-63 (human bone osteosarcoma cells) were cultured in DMEM without phenolred, supplemented with 10% fetal bovine serum (FBS), 100 U mL^-1^ penicillin and streptomycin, 1×GlutaMax (Gibco, Life Technologies, Carlsbad, California), 1×sodium pyruvate (Gibco, Life Technologies, Carlsbad, California) at 37°C (5% CO_2_, humidified atmosphere) according to standard cell culture protocols. MC3T3-E1 (mouse osteoblastic cell line) were cultured in αMEM, supplemented with 10% fetal bovine serum (FBS), 100 U mL^-1^ penicillin and streptomycin, 1% NEAA (Gibco, Life Technologies, Carlsbad, California) at 37°C (5% CO_2_, humidified atmosphere), according to standard cell culture protocols.

12 h before the incubation with nanoparticles, the cells were trypsinized and seeded in cell culture dishes with 5∙10^4^ cells per well in 0.5 mL medium. The incubation with either nanoparticles (Ca/PEI/R-PE) or dissolved R-PE protein was carried out as follows. The particle dispersion (CaP/PEI/R-PE) was added to the growth medium in the ratio of 1:11 (50 μL to 500 μL). This gave a concentration of 2.06×10^8^ nanoparticles per mL, 1.13×10^8^ nanoparticles per well and about 2260 nanoparticles per cell. As control, cells were either incubated with dissolved protein alone (R-PE; 443 μg mL^-1^; 50 μL) or left untreated. After 3 or 6 h of incubation, the cell culture medium was removed and the cells were washed three times with Dulbecco's phosphate-buffered saline (DPBS). After this, only nanoparticles and proteins that were either taken up by the cells or strongly adsorbed on the cell surface remained. The cells were fixed with 4% (w/v) para-formaldehyde for immunofluorescence staining. For live cell imaging experiments, the cells were seeded on 8-well chambered cell culture slides (Falcon™) and incubated with CaP/PEI/R-PE nanoparticles as above. After 6 h of incubation, the cells were washed with pre-warmed (37°C) DPBS and supplied either with fresh medium alone (control) or medium containing 100 nM Bafilomycin A1. The R-PE intensity was then monitored by live cell imaging over a period of 20 h.

### Immunofluorescence

Cells were fixed with 4% (w/v) para-formaldehyde for 20 min, washed twice with DPBS and permeabilised using 0.1% (v/v) Triton X-100 in DPBS for 10 min. For indirect immunofluorescence, samples were washed with DPBS and incubated in blocking solution (3% (v/v) BSA, 0.1% (v/v) Triton X-100, 0.1% (v/v) saponin) for 1 h, followed by incubation with the indicated primary antibodies for 1 h in blocking solution. The cells were washed three times with 0.1% (v/v) Triton X-100 in DPBS and incubated with the fluorescently labelled secondary antibody and Hoechst33342 in blocking solution for 1 h. After washing with 0.1% (v/v) Triton X-100 in DPBS and DPBS alone, the coverslips were mounted on glass slides using ProLong Gold antifade reagent (Thermo Fisher Scientific). For staining of filamentous actin, the fixation, washing and permeabilisation steps were the same as described above. Afterwards, the cells were blocked with 1% (v/v) BSA, 0.1% (v/v) Triton X-100, and 0.1% (v/v) saponin for 30 min, followed by incubation with 1 unit of Alexa Fluor® 660 Phalloidin (200 U mL^-1^) per coverslip in blocking solution for 20 min. Cells were washed three times with 0.1% (v/v) Triton X-100 and stained with DAPI in DPBS (300 nM) for 5 min. Then, the coverslips were washed with DPBS and mounted as described above.

### Microscopy and image analysis

Phase contrast and wide field fluorescence microscopy was performed on a Keyence Biorevo BZ-9000 (Osaka, Japan), equipped with filters for DAPI (EX 360/40, DM 400, BA 460/50), TRITC (R-PE; EX 540/25, DM 565, BA 605/55) and Cy5 (Alexa Fluor® 633 phalloidin; EX 628/40, DM 660, EM 692/40). Images were taken with an S Plan Fluor ELWD 40×/0.60 air objective (Nikon, Japan). Confocal laser scanning microscopy (CLSM) was performed on a TCS SP5 AOBS system equipped with PMT detectors as well as sensitive HyD detectors (Leica Microsystems). The laser lines used for excitation were Diode 405 nm (DAPI; detection range 410–460 nm), DPSS 561 nm (R-PE; detection range 570–620 nm), and HeNe 633 nm (Alexa Fluor® 633; detection range 640–720 nm). Images were acquired with an HCX PL Apo 63×/1.4 oil objective.

For the visualization of the distribution of the fluorescent protein within early endosomes (EEA1) and lysosomes (Lamp1), three independent cell uptake experiments were performed. 30 images per well were taken. The images were processed using the ImageJ software [55] and Photoshop (Adobe Photosystems). Automated image analysis was performed with CellProfiler [56] using a self-written pipeline that gave the percentage of R-PE vesicles co-localized with either Lamp1 or EEA1. Generation of graphs and statistical analysis were performed with GraphPad Prism (Graph Pad Software).

### Bafilomycin A1 chase and time-lapse measurements

The lysosomal degradation chase of nanoparticle-transported R-PE was validated by Bafilomycin A1 (BafA1) treatment. In an eight-chamber well (Falcon), 2.0×10^4^ cells/well were seeded in 400 μL DMEM (1×) supplemented with 10% FBS and 100 U mL^-1^ Penicillin and Streptomycin each and incubated for 24 h at 37°C, 5% CO_2_. Then, 20 μL of a dispersion of CaP/PEI/R-PE nanoparticles were added to the first row of the eight-chamber well, while 20 μL of dissolved R-PE (443 μg mL^-1^) were added to the second row. This gives a concentration of 1.08×10^8^ nanoparticles per mL (dilution 1:21), 4.53×10^7^ nanoparticles per well and about 2,260 nanoparticles per cell. After 6 h of incubation, the cells were washed with pre-warmed (37°C) DPBS twice and the lysosomal degradation chase was started by adding fresh (1×) DMEM containing 100 nM BafA1 in final to two wells of the first and second row of the eight-chamber well, while the remaining wells received (1×) DMEM without BafA1. Cells were imaged with a Keyence Biorevo BZ-9000 microscope (Osaka, Japan) and an air objective S Plan Fluor ELWD 40×/0.60 OFN22 Ph2 WD 3.6–2.8 (Nikon, Japan). Images were taken every 30 min over 24 h. For each well three positions of one well were recorded in three independent experiments. The red fluorescence of the cells was analysed manually in each image with the same illumination parameters. At least 100 cells were counted for each image. The generated data were analysed with the statistical analysis software Prism.

## Results

The red fluorescent phycobilliprotein phycoerythrin (R-PE) is a multimeric protein (240 kDa) that consists of several disk-like subunits containing up to 10 phycoerythrobillin chromophores which are responsible for its fluorescence [57]. This protein is used in immunoassays like fluorescence-assisted cell sorting (FACS), flow cytometry, multimer/tetramer applications, or conjugate labelling chemistry [58–62]. It can also produce singlet oxygen after radiation and might be used in photodynamic therapy (PDT) [63]. It was also shown to be toxic for some cell lines by inducing apoptosis by arresting the cell cycle in the S phase [63]. Its auto-fluorescence (bands at 499, 565 nm and a shoulder at 545 nm) was used here to track the protein alone and together with the nanoparticles. The fluorescence also served as proof that no degradation or damage of its functional integrity had occurred in the endolysosomal system.

**Fig 1:**
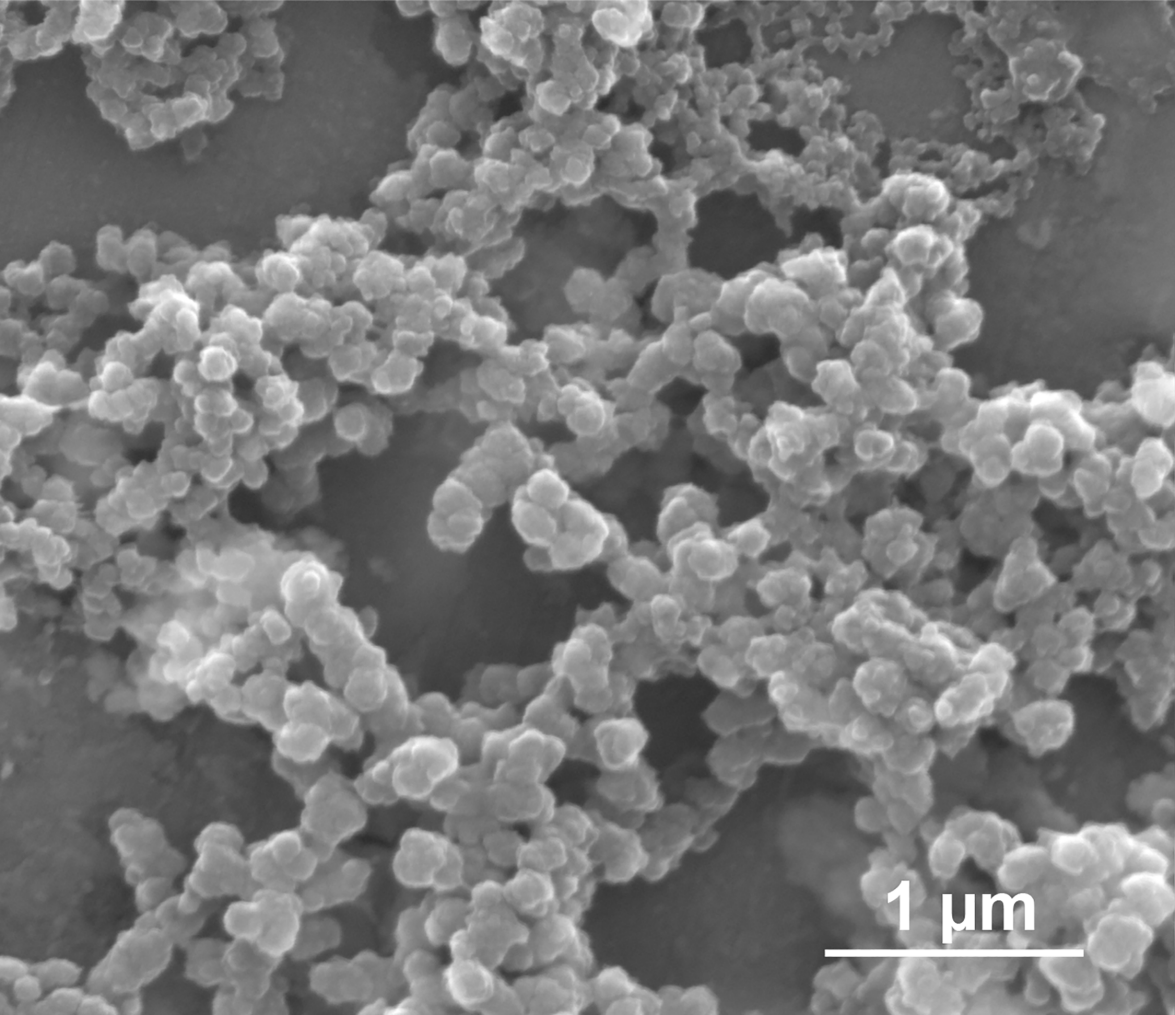
Representative scanning electron micrograph of R-PE-loaded calcium phosphate nanoparticles.

Calcium phosphate nanoparticles were first coated with the cationic polyelectrolyte PEI and then loaded with phycoerythrin (R-PE). The particle diameter was determined by scanning electron microscopy (SEM) with 150 nm (Fig 1). The fact that the particles consisted of calcium phosphate was proven by energy-dispersive X-ray spectrometry (EDX), as shown by the presence of calcium, phosphate, and oxygen, recorded on SEM images. The particles are X-ray amorphous, but we have found crystalline domains in similar calcium phosphate nanoparticles by high-resolution transmission electron microscopy in an earlier study [64].

The dispersion state of particles cannot be derived from scanning electron microscopy because the removal of the solvent and subsequent drying usually causes agglomeration, as seen in Fig 1. Therefore, complementary methods that probe the particle diameter in dispersion are necessary. Dynamic light scattering (DLS) showed that the nanoparticles were well dispersed in water. The average particle diameter was 541 nm, indicating a moderate degree of agglomeration of the individual particles (150 nm) by comparison with the SEM data. The particles were slightly negatively charged (zeta potential -10 mV), indicating a charge reversal of the originally cationic CaP/PEI nanoparticles (zeta potential +22 mV) by the adsorption of negatively charged R-PE (isoelectric point at pH = 4.25 [60]).

**Fig 2:**
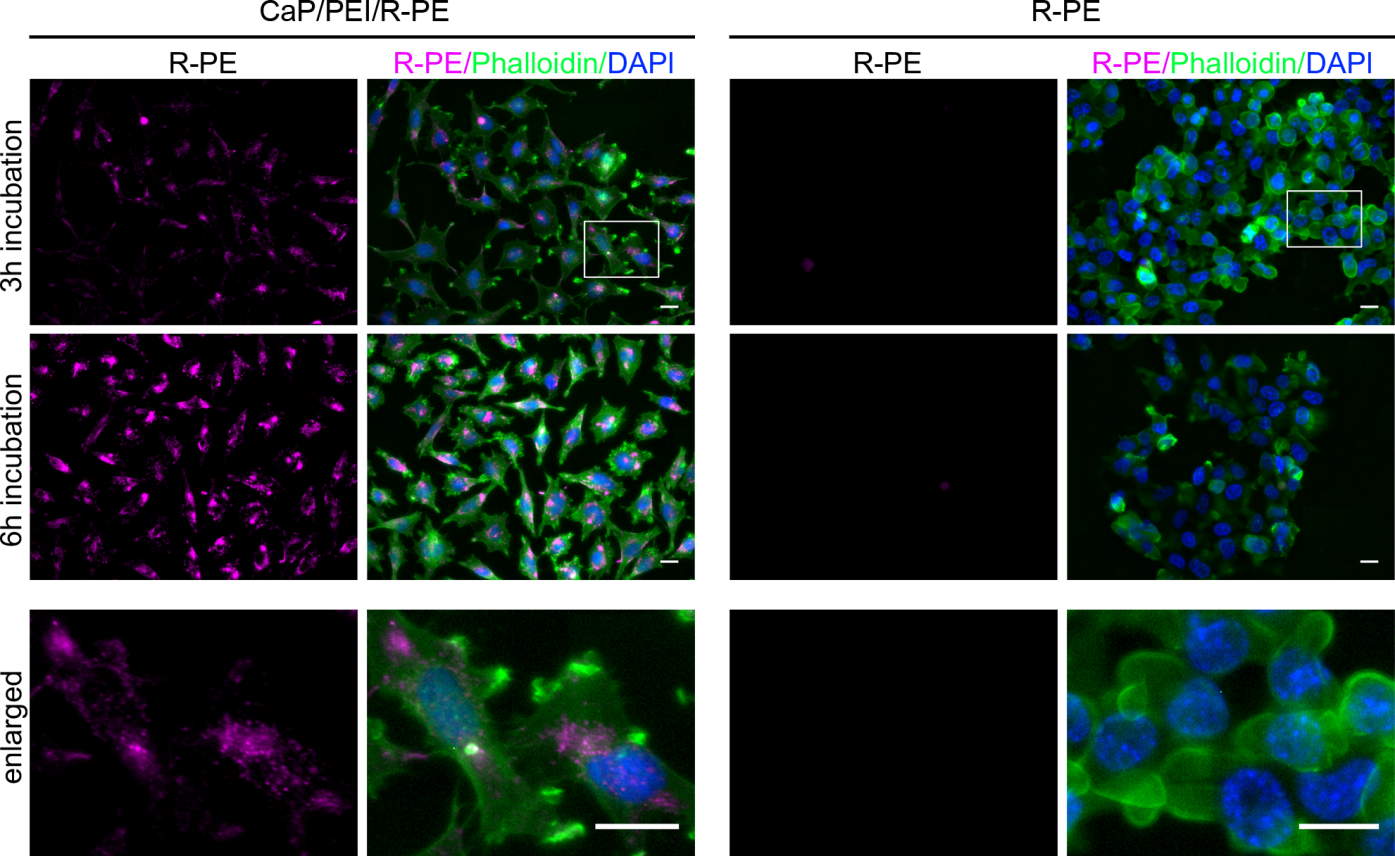
Loading in nanoparticles enables R-PE to enter cells. HeLa cells were incubated with R-PE-loaded nanoparticles (CaP/PEI/R-PE) or dissolved R-PE for 3 h (**top row**) or 6 h (**center row**). Cells were fixed and stained with phalloidin (green; actin filaments) and DAPI (blue; nucleus). **Bottom row:** Magnification of the upper images (white boxes; top row): 3 h incubation R-PE/Phalloidin/DAPI for CaP/PEI/R-PE nanoparticles (left) and 3 h incubation R-PE/Phalloidin/DAPI for R-PE alone (right). All scale bars are 20 μm.

HeLa, HEK293T, MG-63, and MC3T3 cells were incubated with the R-PE-loaded nanoparticles, respectively, and in parallel with the dissolved protein alone at the same protein concentration for 3 and 6 h. All cell lines strongly took up R-PE together with the nanoparticles with an increasing amount from 3 to 6 h. The results for HeLa cells are shown in Fig 2, with equivalent results obtained for the other three cell lines. The protein was not able to cross the cell membrane alone for any cell line, corroborating earlier results on the nanoparticle-mediated uptake of (bio)molecules by cells [3, 11, 29, 40]. The nanoparticle-treated cells did not exhibit any adverse effects due to the presence of calcium or polyethylenimine (PEI), probably due to the low dose (about 1.1 g μL^-1^ calcium phosphate in the well). The concentration of polyethyleneimine on the purified nanoparticles cannot be directly determined, but based on earlier quantitative analyses of polymer-coated nanoparticles [65], we estimate its concentration with 1/10 of the calcium phosphate, *i*.*e*. to about 0.1 μg mL^-1^. This is well below the concentration where cytotoxic effects of PEI have been observed [66]. However, some toxicity of the dissolved R-PE alone was observed for HeLa and HEK293T cells (Fig 2, control experiment), in accordance with earlier observations by Tan *et al*. [63]. Based on our observations we can conclude that the toxicity of R-PE is probably based on an effect occurring from outside the cell. In contrast, MG-63 and MC3T3 cells were less sensitive to R-PE (data not shown).

**Fig 3:**
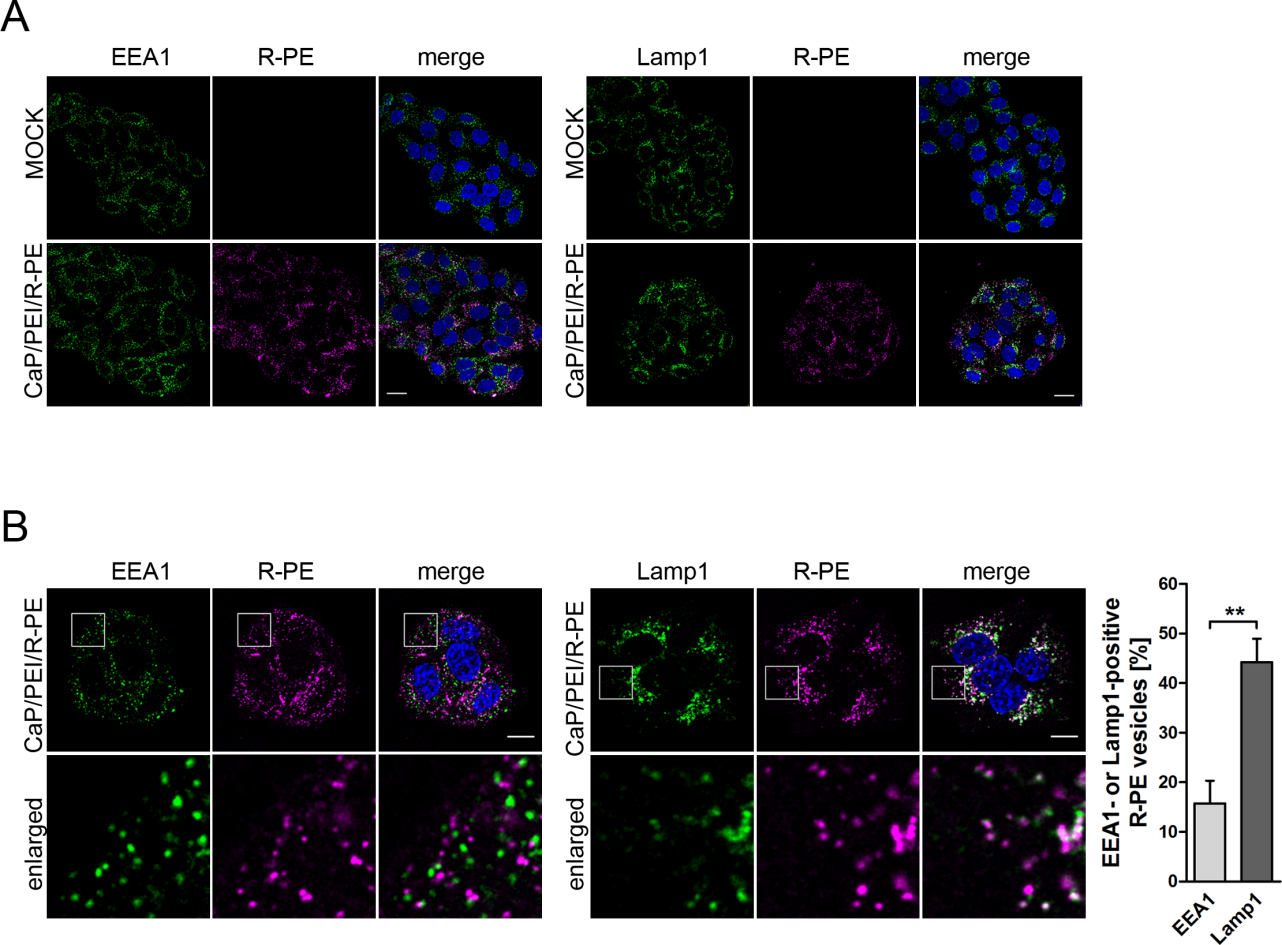
CaP/PEI/R-PE nanoparticles enter HeLa cells and co-localize with early endosomes and lysosomes. (**A**) Confocal laser scanning microscopy on HeLa cells after 6 h of incubation with either CaP/PEI/R-PE nanoparticles or untreated, followed by washing with PBS, fixation and staining with EEA1 (green) or Lamp1 (green) and Hoechst33342 (blue). The overall pattern of early endosomes or lysosomes is not affected by incubation with CaP/PEI/R-PE nanoparticles. Scale bar, 20 μm. (**B**) Higher resolution images to analyse the co-localization of R-PE with EEA1 or Lamp1. HeLa cells were treated and processed as in (**A**). An enlargement of the boxed area is shown. Diagram on the right: Quantification of R-PE vesicles that are positive for EAA1 or Lamp1. Data represent mean ± SD from three independent experiments (student’s t-test). **, *p*<0.01. Scale bar 10 μm.

To further elucidate the intracellular localization of the nanoparticles loaded with R-PE after uptake into HeLa cells, we carried out confocal laser scanning microscopy after 6 h of incubation and subsequent immunostaining for the early endosome-associated protein 1 (EEA1) or the lysosome-associated membrane protein 1 (Lamp1) (Fig 3). The uptake of nanoparticles with R-PE did not change the overall pattern of the endolysosomal system compared to untreated cells (Fig 3A). The R-PE molecules were found both in the early endosomes and lysosomes (Fig 3B), supporting the earlier observation that calcium phosphate nanoparticles are taken up by endocytosis, followed by fusion of the endosome with the degrading lysosome [47]. Endosome-to-lysosome processing can occur within several minutes up to 3 h [67]. Quantitative analysis of the confocal images showed that about 15% of the R-PE vesicles co-localized with the early endosome marker EEA1 and about 45% of the R-PE vesicles were found in lysosomes as shown by co-localization with Lamp1 (Fig 3B). Strikingly, the red fluorescence from R-PE always occurred concentrated to vesicular structures (*i*.*e*. inside endosomes/lysosomes) and not widely distributed across the cell (*i*.*e*. inside the cytoplasm).

No significant amount of R-PE was found inside the cell if the protein was given to the cells without nanoparticles, indicating the need for a nanoparticle to act as transporter across the cell membrane. However, there was only little evidence for R-PE not co-localized with early endosomes or lysosomes in HeLa cells, indicating that it did not escape the lysosome without irreversible damage to its structure. This is also shown by the fact that the red fluorescence from R-PE always occurred concentrated to small areas (*i*.*e*. inside the endosomes/lysosomes) and not widely distributed across the cell (*i*.*e*. inside the cytoplasm).

**Fig 4:**
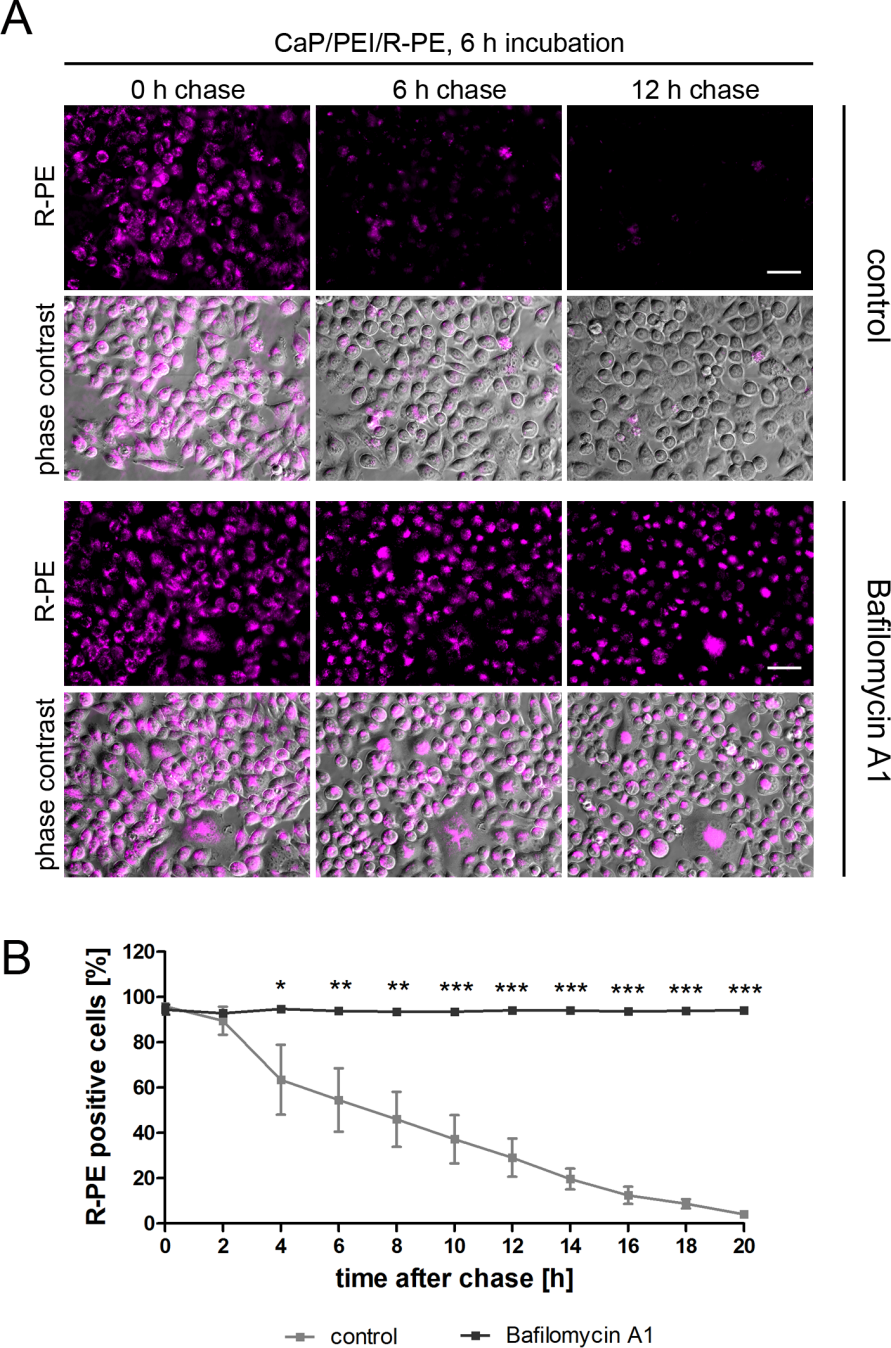
CaP/PEI/R-PE nanoparticles are degraded in lysosomes after uptake into HeLa cells. (**A**) R-PE signal persists after inhibition of lysosomal degradation. HeLa cells were incubated with CaP/PEI/R-PE nanoparticles for 6 h, washed and chased for the indicated time either in the presence of Bafilomycin A1 or without any additive (control). The fluorescence signal of R-PE is shown alone or merged with phase contrast to visualize the cells. Scale bar 50 μm. (**B**) Quantification of R-PE positive cells from three independent experiments. Data represent mean ± SD (student’s t-test). *, *p*<0.05, **, *p*<0.01, ***, *p*<0.001.

To examine if R-PE is able to escape from lysosomes at later time points, we monitored its fate over time by live cell imaging, first with HeLa cells. After 6 h of incubation with R-PE-loaded nanoparticles, cells were washed and further cultivated in nanoparticle-free medium. The red fluorescence of R-PE vanished over time (Fig 4). At no time point, a clear cytoplasmic distribution of R-PE was observed. This result strongly suggests that R-PE is degraded in lysosomes. To verify this assumption and to exclude fast escape into the cytoplasm and fast degradation there, we performed the experiment of chasing the HeLa cells in nanoparticle-free medium with Bafilomycin A1. This is a specific inhibitor of vacuolar-type H^+^-ATPase that inhibits the acidification and protein degradation in lysosomes of cultured cells [68]. Indeed, the intrinsic fluorescence of R-PE was preserved over the whole observation time in vesicular structures (Fig 4).

**Fig 5:**
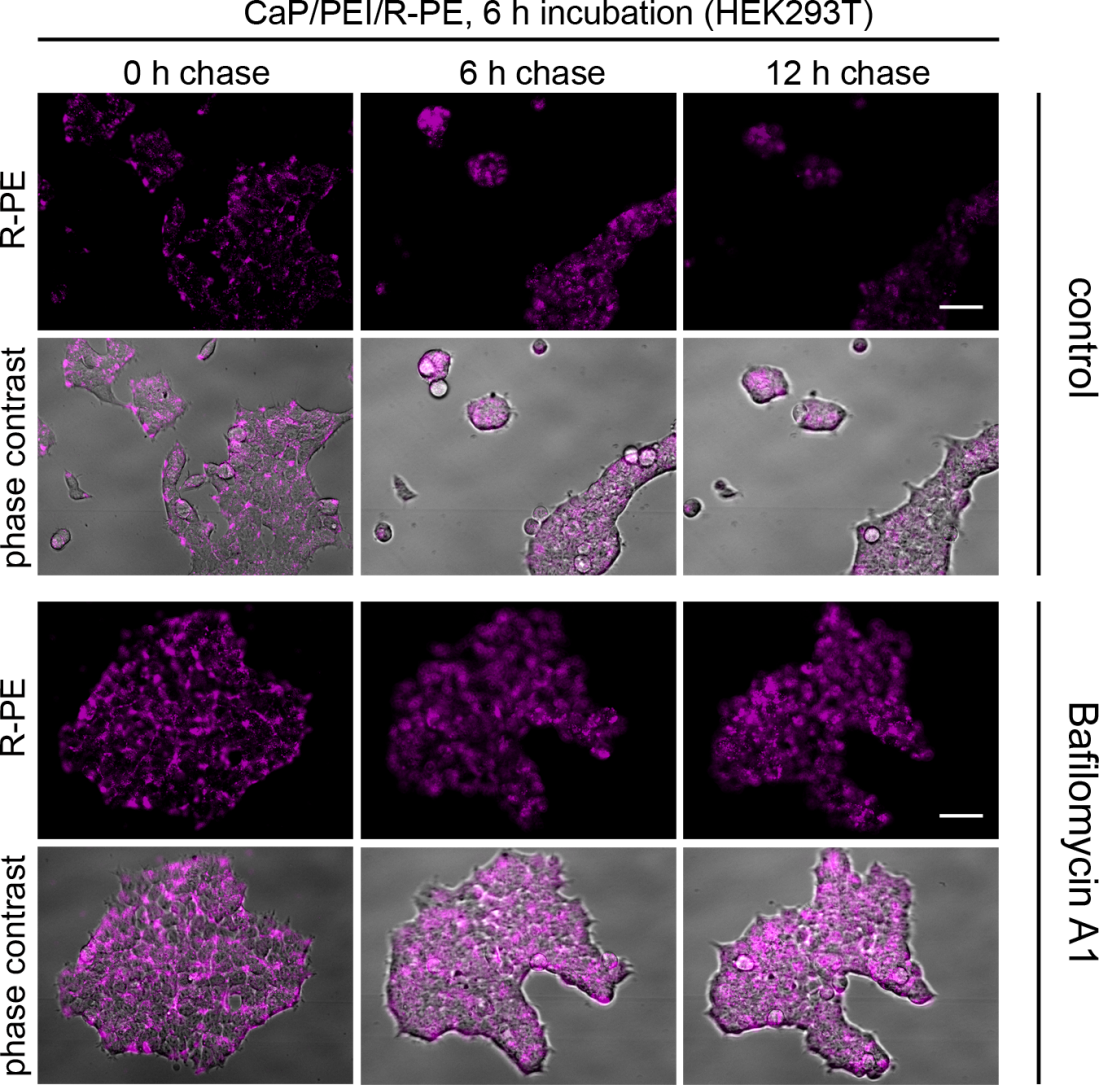
CaP/PEI/R-PE nanoparticles are not degraded in lysosomes after uptake into HEK293T cells. There is no degradation of R-PE after cellular uptake as indicated by the persistent fluorescence (control). HEK293T cells were incubated with CaP/PEI/R-PE nanoparticles for 6 h, washed and chased for the indicated time either in the presence of Bafilomycin A1 or without any additive (control). The fluorescence signal of R-PE is shown alone or merged with phase contrast to visualize the cells. Scale bar 50 μm.

**Fig 6:**
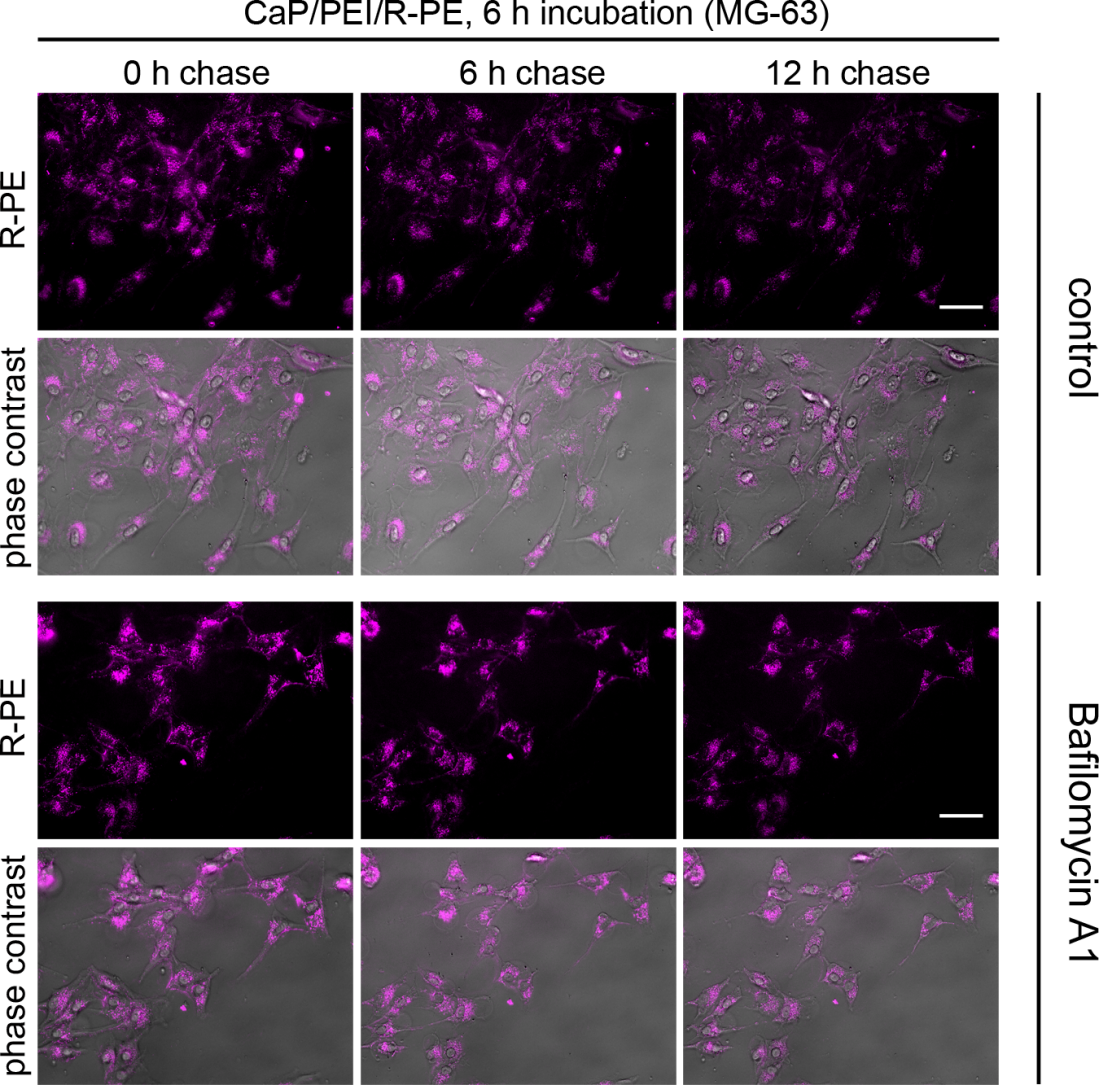
CaP/PEI/R-PE nanoparticles are not degraded in lysosomes after uptake into MG-63 cells. There is no degradation of R-PE as indicated by the persistent fluorescence intensity (control). MG-63 cells were incubated with CaP/PEI/R-PE nanoparticles for 6 h, washed and chased for the indicated time either in the presence of Bafilomycin A1 or without any additive (control). The fluorescence signal of R-PE is shown alone or merged with phase contrast to visualize the cells. Scale bar 50 μm.

**Fig 7:**
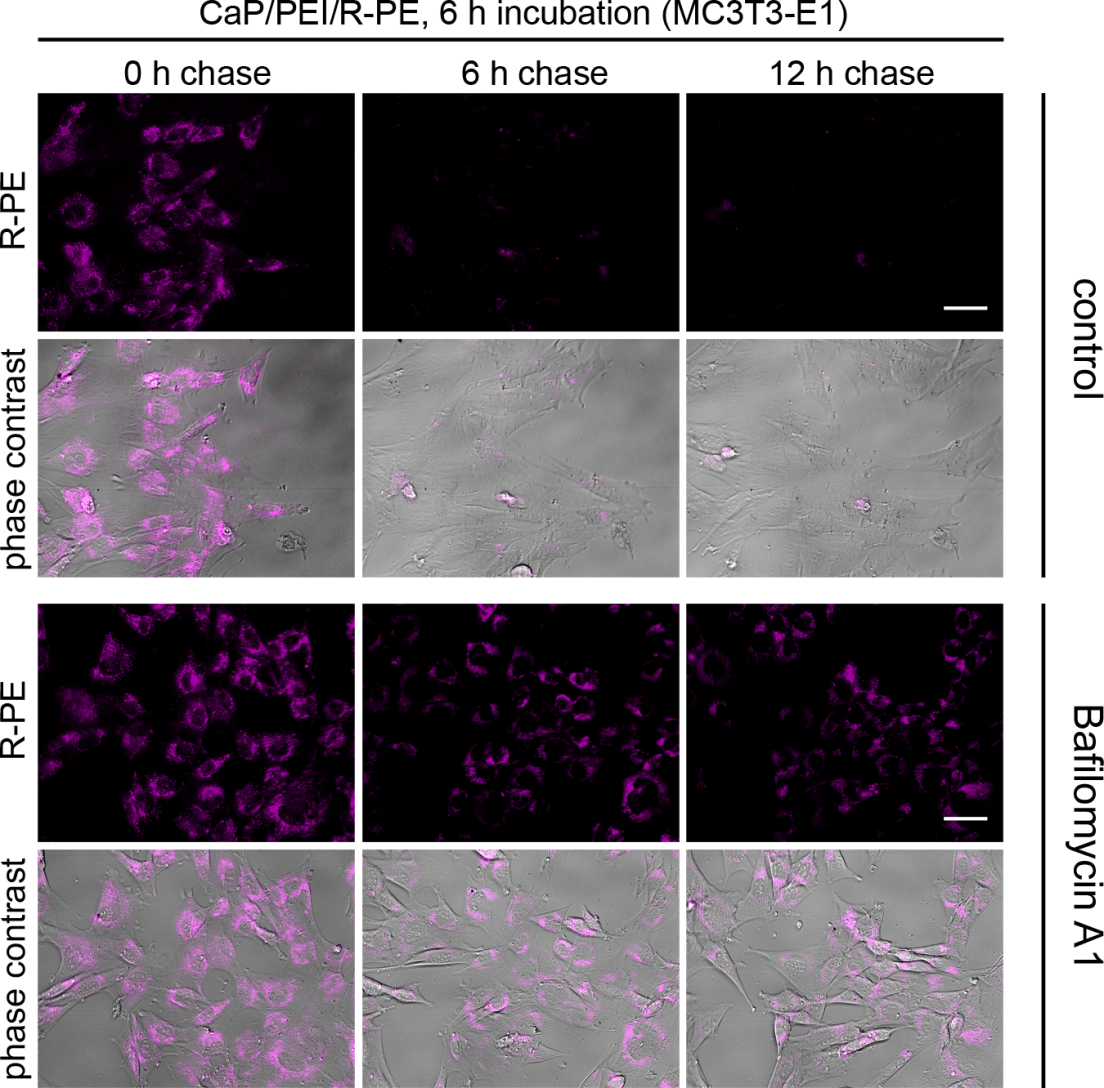
CaP/PEI/R-PE nanoparticles are degraded in lysosomes after uptake into MC3T3 cells. After cellular uptake, R-PE is rapidly degraded as indicated by the vanishing fluorescence intensity (control). MC3T3 cells were incubated with CaP/PEI/R-PE nanoparticles for 6 h, washed and chased for the indicated time either in the presence of Bafilomycin A1 or without any additive (control). The fluorescence signal of R-PE is shown alone or merged with phase contrast to visualize the cells. Scale bar 50 μm.

Bafilomycin chase experiments were also carried out for HEK293T, MG-63, and MC3T3 cell lines under the same conditions and with the same calcium phosphate nanoparticles, loaded with R-PE. As stated above, the particles were able to transport the R-PE into cells of all four cell lines (Figs 5–7). Inside HEK293T cells and MG-63 cells, R-PE was not degraded. In contrast, in MC3T3 cells, the protein was rapidly degraded within 6 h, comparable to HeLa cells. Bafilomycin A1 prevented the degradation of R-PE in all cases, indicating that the degradation occurred by proteolysis.

## Discussion

The delivery of therapeutic molecules with the help of nanoparticles is a major goal of nanomedicine [3, 69, 70]. In gene therapy, this is exploited for transfection and gene silencing [5, 6, 8], in immunology, it is used for vaccination and cell stimulation [71–73], and in tumour therapy, it is used to deliver therapeutic molecules like cytostatics [4, 74, 75]. Typically, a transport of these drugs across the cell membrane is desired, and the functional integrity of the drugs must be assured to preserve the therapeutic effect. For gene delivery and immunology, this concept works as demonstrated by many successful transfection, gene silencing, and immunization studies (see, *e*.*g*., the references cited above). If nucleic acids were degraded completely within the lysosome, they would not be able to exert their genetic effect. However, it has been shown that an early endosomal escape that prevents lysosomal degradation is highly advantageous for an efficient gene transfer [76–78]. Especially the "proton-sponge effect" induced by cationic polyelectrolytes like PEI has been discussed to facilitate the endosomal escape [50, 52, 79–81]. This has also been proposed to be the case for calcium phosphate nanoparticles that increase the osmotic pressure in the lysosome due to dissolution into ions [82].

Red-fluorescing R-PE is taken up by all four investigated cell lines with the help of nanoparticles after 3 to 6 h. After 6 h, the protein is mainly located in early endosomes and lysosomes, as shown for HeLa cells. From earlier studies, we know that the uptake of calcium phosphate nanoparticles into HeLa cells mainly occurs by macropinocytosis and endocytosis [47]. Both pathways first lead into early endosomes which are then fusing with lysosomes where degradation by digesting proteases and an acidic environment occurs [48]. At least in the case of R-PE after uptake by HeLa cells and MC3T3 cells, the endosomal escape by increased osmotic pressure due to the presence of PEI and calcium phosphate does not work.

Thus, for these two cell lines R-PE is not escaping from the lysosomes but undergoes endolysosomal degradation. It has been shown that the fluorescence of R-PE does not significantly change in the pH range between 3.5 and 10 [83]. The pH inside a lysosome is between 4.5 and 5 [84], therefore the autofluorescence of R-PE did not disappear due to the low pH but rather due to a proteolytic decomposition. Furthermore, the calcium phosphate nanoparticle does not dissolve at neutral pH as shown earlier [37, 39]. In contrast, HEK293T cells and MG-63 cells took up R-PE with nanoparticles, but no degradation occurred, even without Bafilomycin. This indicates that the fate of a protein and a nanoparticle depends on the individual cell line. The degradation of R-PE within a few hours in two out of four investigated cell lines points to differences between the delivery of nucleic acids and proteins with the help of nanoparticles [85]. The difference may be due to different degradation mechanisms, *i*.*e*. nucleases in the case of DNA and siRNA and proteases in the case of proteins. This deserves further attention, especially when a nanoparticle-mediated therapeutic delivery of proteins into a cell is desired.

We wish to add that the presented results are only indicative for this special case, *i*.*e*. the transport of R-phycoerythrin by calcium phosphate nanoparticles into these four cell cells. However, the endocytotic uptake of nanoparticles into cells has also been demonstrated many times [8, 41, 42, 45, 46, 86–88], including our comprehensive study with 10 different cell lines and calcium phosphate nanoparticles [82]. Together with earlier proofs for the transport of biomolecule-loaded calcium phosphate nanoparticles by endocytosis into various cell lines [37, 39, 40, 47, 82], it is very likely that the described scenario applies also to other situations where proteins are delivered into cells with the help of nanoparticles.

## Conclusions

The autofluorescent protein R-phycoerythrin is easily taken up by four different cell lines with the help of calcium phosphate nanoparticles, but not in dissolved form without nanoparticles. Our results highlight the fact that following a fluorescent label attached to a biomolecule is not the same as following the protein (and its integrity) itself. A successful nanoparticle-mediated uptake of a labelled protein (and other cargo molecules) does not necessarily mean that the protein is still functional. As the function of a protein inside a cell is often difficult to measure and to quantify, autofluorescent proteins offer an easy way to study the efficiency of new carrier systems for biomolecules.
